# Supplementary material for: P2X7 Is Involved in the Mouse Retinal Degeneration via the Coordinated Actions in Different Retinal Cell Types
Source: Antioxidants (Basel). 2023 Jan 6;12(1):141. doi: 10.3390/antiox12010141 (PMC9854982; doi:10.3390/antiox12010141)
Supplement: Supplementary file 1 [file antioxidants-12-00141-s001.zip › antioxidants-2120965-supplementary.pdf]

Supplementary Table S1: qPCR primers.

	Forward primer	Reverse primer	Gene accession number
IL-1β	TTC AAG GGG ACA TTA GGC AG	TGT GCT GGT GCT TCA TTC AT	NM_008361.4
IL-6	TTC CTC TCT GCA AGA GAC TTC	GGT CTG TTG GGA GTG GTA TC	NM_001314054.1
NLRP3	AGA GAA TGA GGT CCT CTT TAC CAT	AGC CCC GTG CAC ACA ATC	NM_001359638.1
Caspase 3	GAC TGA TGA GGA GAT GGC TTG	TGC AAA GGG ACT GGA TGA AC	NM_001284409.1
Caspase 7	CCC ACT TAT CTG TAC CGC ATG	GGT TTT GGA AGC ACT TGAAGA	NM_007611.2
Caspase 8	AAC TTC CTA GAC TGC AAC CG	TCT CAA TTC CAA CTC GCT CAC	NM_001080126.1
β-actin	CGG GGA CCT GAC TGA CTA CC	AGG AAG GCT GGA AGA GTG C	NM_007393.5
